# Zika virus infection as an unexpected finding in a Leptospirosis patient

**DOI:** 10.1099/jmmcr.0.005033

**Published:** 2016-06-10

**Authors:** Antoine Biron, Cécile Cazorla, Julien Amar, Anne Pfannstiel, Myrielle Dupont-Rouzeyrol, Cyrille Goarant

**Affiliations:** ^1^​ Institut Pasteur de Nouvelle-Caledonie, Medical Virology Laboratory, Nouméa, New Caledonia; ^2^​ Centre Hospitalier Territorial de Nouvelle-Caledonie, Infectious Disease Department, Nouméa, New Caledonia; ^3^​ Centre Hospitalier Territorial de Nouvelle-Caledonie, Intensive Care Unit, Nouméa, New Caledonia; ^4^​ Gouvernement de la Nouvelle-Caledonie, Direction des Affaires Sanitaires et Sociales, Nouméa, New Caledonia; ^5^​ Institut Pasteur de Nouvelle-Caledonie, Arbovirus Research and Expertise Unit, Nouméa, New Caledonia; ^6^​ Institut Pasteur de Nouvelle-Caledonie, Leptospirosis Research and Expertise Unit, Nouméa, New Caledonia

**Keywords:** leptospirosis, zika virus, shock syndrome, amoxycillin, differential diagnosis, dual infection

## Abstract

**Introduction:**

Areas where leptospirosis and arboviruses are endemic largely overlap in the tropics. However, the number of arbovirus infections is usually much higher. The initial clinical presentation can be highly confusing; therefore, laboratory confirmation is key to an accurate diagnosis.

**Case Presentation:**

A 19–year–old man presented to a peripheral health centre with an acute febrile illness. Dengue was initially suspected, but the patient deteriorated to a shock syndrome. Leptospirosis as well as a co-infection with Zika virus were both confirmed in the laboratory, the latter being clinically masked in this dual infection.

**Conclusion:**

This case highlights the importance of not only considering the differential diagnosis of acute febrile syndromes, but also to consider the possibility of dual infections in the context of global spread of arboviruses. The specific context of travellers returning from endemic areas and pregnant women is also highlighted and discussed.

## Introduction

In the last few years, an unprecedented wave of mosquito-borne viral infections has hit the Pacific Island Countries and Territories (Roth *et al.*, 2014), and has also spread globally, putting new naïve populations at risk (Noel & Rizzo, 2014). As a result, a high level of awareness of arboviral diseases has reached the medical and public health communities leading to increased surveillance. In some tropical settings (e.g. in New Caledonia), all requests for laboratory diagnosis of any arboviral infection (dengue, chikungunya or Zika) in a hospitalized patient are systematically tested for all three viruses, as part of the public health surveillance scheme. A similar high awareness is not present for leptospirosis, a zoonotic bacterial disease, which is one of the possible differential diagnosis of acute febrile syndromes. We report a case of severe leptospirosis (with an initial suspicion of dengue) that was co-infected with Zika virus, emphasizingthe risk of *Leptospira* and arbovirus dual infections, already reported before (Sharp *et al.*, 2012).

## Case report

**Fig. 1.**
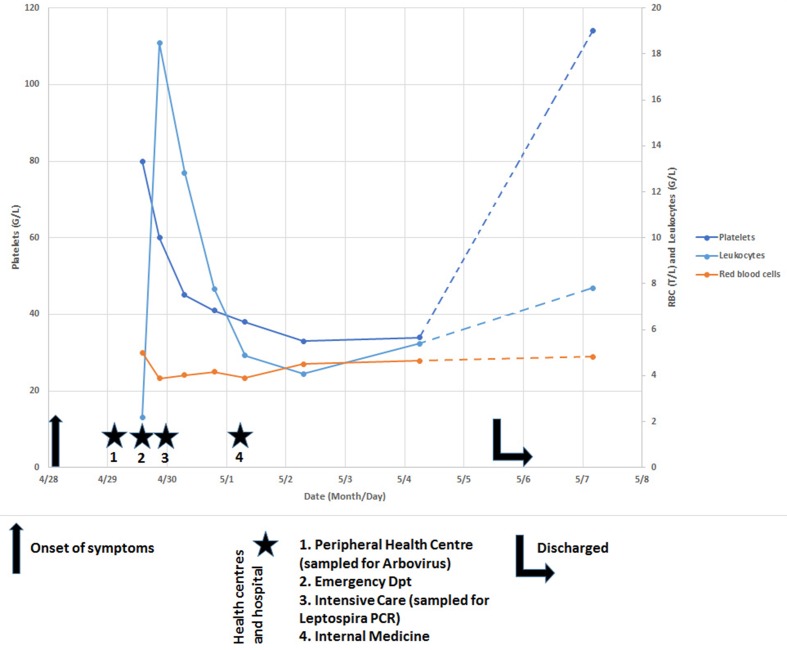
Blood cell counts and timeline of medical history.

A 19-year–old male with previous good health and no significant past medical history, presented to a peripheral health centre 24 h after the onset of a febrile flu-like syndrome with fever, myalgia and painful cough. A suspicion of dengue led to a request for a laboratory diagnosis, and the patient was referred to the emergency department of New Caledonia central hospital, where he arrived 36 h after the onset of fever (Fig. 1). The pulmonary auscultation was normal, but the hemodynamic condition was unstable. Upon questioning, the patient reported a swim in a river on the North-East coast of New Caledonia two weeks before, a known exposure risk for leptospirosis in the Pacific (Berlioz-Arthaud *et al.*, 2007). A blood culture and a *Leptospira* PCR were requested. The hemodynamic condition rapidly deteriorated, and the patient was transferred to the Intensive Care Unit, where he received noradrenalin, fluid infusion and was treated with Ceftriaxone and Gentamycin for a sceptic shock. Blood analysis revealed a thrombocytopenia 80×10^9^ L^−1^, elevated C-Reactive Protein (CRP 241.5 mg L ^−1^), Procalcitonin (PCT, 60.9 ng mL^−1^), Lactate (3.62 mmol L^−1^) and Lactate Dehydrogenase (LDH, 256 UI L^−1^).

The next day, *Leptospira* PCR (Stoddard *et al.*, 2009 returned a positive result with a quantified 192 bacteria/ml. The *Leptospira lfb1* sequence used to identify the putative infecting serovar (Perez & Goarant, 2010) pointed to serogroup Icterohaemorrhagiae. The patient clinically improved, leading to discontinuation of noradrenaline support. Similarly, Ceftriaxone and Gentamycin were changed to Amoxycillin upon receipt of the positive *Leptospira* PCR result. The patient was transferred the next day to the Infectious Disease Department where Amoxycillin was continued. The laboratory blood results normalized, and patient was disharged after a week. However, there was persistent thrombocytopenia (34×10^9^ L^−1^). At the time of discharge, the Zika RT-qPCR on the sample collected at first medical visit at the peripheral health centre returned a positive result (on both RNA targets(Lanciotti, 2008)), with an estimated 2×10^4^ to 1.5×10^5^viral RNA/ml. A full blood count showed a return to subnormal platelet count (114×10^9^ L^−1^) two days later. The timeline of medical history is summarised in Fig. 1, the relevance of each symptom to both infectious agents is considered in Table 1.

**Table 1.** Clinical symptoms and laboratory results and their relevance to leptospirosis and Zika virus

Parameter	Leptospirosis	Zika Virus
Fever >38.5°C	+++	+
Headache	++	+
Myalgia	+++	+/-
Initial Leukocytopenia	++	+
Later Leukocytosis	+++	-
Thrombocytopenia	+++	+
Low RBC and hemoglobin	+++	-
High CRP	+++	-
High PCT	+++	-
Increased Lactates & LDH	+++	-

-, +/-, +, ++ and +++ indicate the relavance for each parameter in increasing order (- not relevant; +++ fully relevant)

## Discussion

Leptospirosis is endemic in New Caledonia, and the medical community is highly aware of this bacterial zoonosis. Epidemiological criteria are systematically considered for leptospirosis suspicion. However, because of unprecedented epidemics of arbovirus in the Pacific, and the larger size of outbreaks, arboviral diseases are often considered high up in the list of differential diagnoses of acute febrile syndromes. The patient presented early but probably failed to mention freshwater swimming, which is a well known risk factor in New Caledonia, and so leptospirosis was initially not considered in the differential diagnosis. His condition rapidly deteriorated to a septic shock with laboratory tests indicating a bacterial infection (CRP and PCT). Quantitative PCR and genotyping revealed a leptospiraemia caused by a strain from serogroup Icterohaemorrhagiae. The rapid presentation and antimicrobial treatment probably contributed to prompt recovery (Tubiana *et al.*, 2013). Zika virus co-infection was discovered thanks to the systematic laboratory diagnosis of Zika, chikungunya and dengue, as part of the New Caledonian surveillance system but only when the patient was ready to be discharged. This virus probably contributed little to the patient’s clinical presentation although it possibly contributed to the thrombocytopenia (Table 1).

Although public and medical awareness are essential to arboviral diseases control and despite the tremendous media coverage of Zika and other arboviral infections in the last few years, careful differential diagnosis of acute fevers is essential during arbovirus outbreaks (Ellis & Imrie, 2008) to exclude leptospirosis and other antibiotic-treatable bacterial infections. This would help to minimize the rapid evolution to severe complications in leptospirosis cases.

The case reported here shows that Zika virus infection can easily be masked in dual infections by another, more symptomatic, viral or bacterial infection (Dupont-Rouzeyrol *et al.*, 2015) because it is associated with few symptoms. Both leptosirosis and Zika virus might also be present in travellers returning from the tropics, and this should always be considered in such patients (Arcilla *et al.*, 2012 , Maria, *et al.*, 2016). This is important because it might not only lead to post-infectious neurological disorders (Cao-Lormeau *et al.*, 2016), but also failure to arrange appropriate follow-up in pregnant women, if it is not diagnosed correctly. The case reported here highlights the importance of accurate diagnosis and management of dual infections with appropriate use of antibiotic therapy. This should especially be considered in returning travellers or when Zika infection is present in pregnant women.
